# Predicting Potential Global Distributions of Two *Miscanthus* Grasses: Implications for Horticulture, Biofuel Production, and Biological Invasions

**DOI:** 10.1371/journal.pone.0100032

**Published:** 2014-06-19

**Authors:** Heather A. Hager, Sarah E. Sinasac, Ze’ev Gedalof, Jonathan A. Newman

**Affiliations:** 1 School of Environmental Sciences, University of Guelph, Guelph, Ontario, Canada; 2 Department of Geography, University of Guelph, Guelph, Ontario, Canada; Instituto de Agricultura Sostenible (CSIC), Spain

## Abstract

In many regions, large proportions of the naturalized and invasive non-native floras were originally introduced deliberately by humans. Pest risk assessments are now used in many jurisdictions to regulate the importation of species and usually include an estimation of the potential distribution in the import area. Two species of Asian grass (*Miscanthus sacchariflorus* and *M. sinensis*) that were originally introduced to North America as ornamental plants have since escaped cultivation. These species and their hybrid offspring are now receiving attention for large-scale production as biofuel crops in North America and elsewhere. We evaluated their potential global climate suitability for cultivation and potential invasion using the niche model CLIMEX and evaluated the models’ sensitivity to the parameter values. We then compared the sensitivity of projections of future climatically suitable area under two climate models and two emissions scenarios. The models indicate that the species have been introduced to most of the potential global climatically suitable areas in the northern but not the southern hemisphere. The more narrowly distributed species (*M. sacchariflorus*) is more sensitive to changes in model parameters, which could have implications for modelling species of conservation concern. Climate projections indicate likely contractions in potential range in the south, but expansions in the north, particularly in introduced areas where biomass production trials are under way. Climate sensitivity analysis shows that projections differ more between the selected climate change models than between the selected emissions scenarios. Local-scale assessments are required to overlay suitable habitat with climate projections to estimate areas of cultivation potential and invasion risk.

## Introduction

Plant species are often introduced to new regions through human intervention. Plants that were introduced historically for medicinal, agricultural, or horticultural uses compose a large proportion (>60%) of the currently naturalized angiosperms in the United States and elsewhere [1]. Once established, these species have the potential to become invasive, with subsequent negative ecological and economic effects [2]. Many jurisdictions have introduced weed risk assessment methods to evaluate the risk that deliberately introduced plant species will become invasive in the future (e.g., [3], [4], [5], [6]). However, for species that were introduced prior to widespread use of such methods, their proliferation through increasing horticultural sales or industrial cultivation could increase their risk of escape. Assessing the risks and potential invasive outcomes of such species is therefore important in developing best management practices [7], [8], [9].

Several species of *Miscanthus* have been introduced to novel ranges in North America, Europe, and Scandinavia for both horticultural and agricultural purposes. These are tall, perennial, rhizomatous, C4 grasses native to temperate, humid subtropical, and tropical savannah climates of Asia [10]. *Miscanthus sinensis* Andersson was first introduced to North America as an ornamental plant in the 1890 s; it has since escaped cultivation in the northeastern United States and is considered invasive in some states [11]. Less is known about the first introduction of *M. sacchariflorus* (Maxim.) Franch. as an ornamental plant in North America, but escaped specimens were noted in the mid-western United States by 1950 [12], and the first escaped specimens in Ontario, Canada, were collected in 1952 [13]. These two species produce a sterile hybrid *M. x giganteus* J.M. Greef & Deuter ex Hodkinson & Renvoize that has been encountered infrequently in the wild [14]. *M. sinensis* has become a very popular ornamental plant in areas of the United States and Canada [15], and all three species are of interest as potential biofuel crops (e.g., [16], [17], [18]). Breeding programs for horticultural and agricultural improvement could enhance the potential of these species to be invasive [19], [11].

The role of weed risk assessment is to evaluate the risk of escape from cultivation and the extent of possible economic and environmental damage [4]. Thus, many assessments include estimating the potential climate suitability of the risk assessment area for candidate species for import (e.g., [3], [4], [5], [6]) because climate is a good predictor of plant distributions [20], [21]. Climate suitability provides a coarse-scale estimate of a species’ distribution while ignoring factors such as substrate geology, biotic interactions, and infrastructure, which affect regional habitat suitability.

Various methods have been used to estimate potential species distributions, for example, plant hardiness zones [22], [6], climate regions occupied [23], and a wide range of bioclimatic and niche models (e.g., [24], [25], [9]). The latter use detailed information on temperature and moisture in the species’ native range to determine areas with potentially suitable climate for population persistence. Estimating species preferences for certain growing conditions can be difficult, leading to uncertainty in model parameterization. However, model sensitivity to the choice of parameter values is seldom evaluated (but see [26], [27]), but is important in understanding which parameters have the greatest effect on model output and therefore should be estimated most carefully or where results should be treated most cautiously. Such information can be used in interpreting model output and in directing future research to improve model reliability.

Estimating species’ potential ranges under projected climate change is also a priority. This work is of particular concern for species of agronomic and horticultural importance, as well as for invasive and potential pest species [28]. Shifts in distributions of agronomic and horticultural species are likely to keep pace with climate change if people continue to plant them where conditions are suitable [29]. Distribution shifts of pests and invaders could also be favoured under climate change because these species tend to have wide physiological tolerances and traits that allow them to take advantage of long-distance (human-assisted) dispersal vectors [30]. Previous assessments of climatic suitability indicate that the modelled distributions differ depending on the choice of climate change model and scenario (e.g., [31], [32]). Comparing multiple models and climate change scenarios therefore allows assessment of the sensitivity of results to these choices.

The purpose of this analysis was threefold. First, we estimated the current potential global distributions of *M. sacchariflorus* and *M. sinensis* using a commonly employed niche model. Second, we evaluated the model sensitivity to estimate which parameters are most critical and whether this differs between the species. Third, we examined how the potentially suitable area is projected to change under future climates, as well as how sensitive these results are to the selection of climate model and emissions scenario. The resulting potential distributions indicate areas that might be suitable for horticultural or agricultural cultivation of these species, but also susceptible to their invasion, should they escape.

## Methods

We estimated the current global native and introduced distributions of *M. sacchariflorus* and *M. sinensis* using species occurrence data from the Global Biodiversity Information Facility (GBIF; www.gbif.org), botanical garden records, and a literature search of agronomic trials. All data were sorted to remove duplicate records and were separated based on whether geolocation information (latitude and longitude) was provided or whether we could geocode the record using information such as street name, city, county, or region (“inferred” location). Records that did not provide location information below the country level were omitted.

We used the native distribution data to model the potential climatic range of each species using the CLIMEX Compare Locations model [33], [34]. We used 0.5° world grid climate data provided with the software (from the Climate Research Unit at Norwich, UK [35]). CLIMEX assumes that the geographical distribution of the species is limited by climate; it does not generally account for biotic interactions [36] or substrate type. It calculates an annual growth index based on the species’ fitted temperature and moisture response functions, as well as four stress indices (hot, cold, dry, wet, and their combinations) to calculate an ecoclimatic index (EI). The EI is an estimate of a location’s climatic suitability to support a persistent population of the species being modelled [34]. EI ranges from 0 to 100, with 0 meaning the area is not suitable for species persistence, and 100 meaning the climate is optimal for the species year-round. In practice, EI of 100 is only attained for species in stable and ideal climate [34].

For each species, we began with parameter values from the default temperate template and adjusted them iteratively based on the species’ biology until the modelled distributions approximated the native distributions [34]; these included humid subtropical (*M. sacchariflorus, M. sinensis*) and tropical savannah (*M. sinensis*) climates of Asia. The model was then validated by comparison with the observed distribution records in the species’ introduced ranges in Europe and North America. Given the satisfactory fit, no further adjustments were required at this stage for either model.

### Parameter Fitting

#### Temperature and cold/hot stress

Based on their native distributions, both *M. sacchariflorus* and *M. sinensis* are well suited for cold-temperate regions (e.g., [37], [38]). Their percentage shoot emergence at experimental, low temperatures ranges from 10 to 100% at 7 to 15°C [39]. Their *M.* x *giganteus* hybrid shows only minor reduction in leaf photosynthetic capacity when grown at 10°C or 14°C compared to 25°C [40], [41]. Therefore, the limiting low temperature (DV0) and lower optimal temperature (DV1) were set at 5°C and 15°C, respectively, for both species (Table 1). This and the cold stress (below) accounted for the northernmost occurrence of both species in far northeastern China and the southeastern Primorsky Krai region of Russia.

**Table 1:** Fitted parameter values used to generate bioclimatic envelope models of *Miscanthus sacchariflorus* and *M. sinensis* distributions using CLIMEX.

Parameter description	Parameter	*Miscanthus sacchariflorus*	*Miscanthus sinensis*
**Moisture**			
Limiting low moisture^a^	SM0	0.25	0.25
Lower optimal moisture^a^	SM1	0.8	0.8
Upper optimal moisture^a^	SM2	1.2	1
Limiting high moisture^a^	SM3	1.8	2.5
**Temperature**			
Limiting low temperature^b^	DV0	5	5
Lower optimal temperature^b^	DV1	15	15
Upper optimal temperature^b^	DV2	28	30
Limiting high temperature^b^	DV3	32	35
**Cold Stress**			
Cold stress temperature threshold^b^	TTCS	−5	−5
Cold stress temperature rate^c^	THCS	−0.0002	−0.0002
Cold stress degree-day temperature threshold^d^	DTCS	12	14
Cold stress degree-day rate^c^	DHCS	−0.00005	−0.00005
**Heat Stress**			
Heat stress temperature threshold^b^	TTHS	32	36
Heat stress temperature rate^c^	THHS	0.06	0.05
**Dry Stress**			
Dry stress moisture threshold^a^	SMDS	0.1	0.1
Dry stress rate^c^	HDS	−0.02	−0.02
**Wet Stress**			
Wet stress moisture threshold^a^	SMWS	1.8	3
Wet stress rate^c^	HWS	0.02	0.05
**Hot-wet Stress**			
Hot-wet temperature threshold^b^	TTHW	31	-
Hot-wet moisture threshold^a^	MTHW	1	-
Hot-wet stress rate^c^	PHW	0.01	-
**Degree-days**			
Minimum degree days above DV0 to complete one generation^d^	PDD	600	600

a Proportion soil capacity.

b °C.

c Week^−1^.

d °D.

The upper optimal temperature (DV2) and limiting high temperature (DV3) were adjusted based on maximum temperatures occurring in the native region [42] and observations that the experimental optimal temperature for photosynthesis of their hybrid is between 30°C and 35°C [43]. The native distribution of *M. sinensis* extends much further south into tropical regions than does that of *M. sacchariflorus* [37]. Therefore, both DV2 and DV3 for *M. sinensis* were greater than those for *M. sacchariflorus* (Table 1).

For the cold stress index, we used parameters related to overwinter survival (lethal temperatures: cold stress temperature threshold, TTCS, and cold stress temperature rate, THCS) and cold stress affecting metabolism (cold stress degree day threshold, DTCS, and cold stress degree day rate, DHCS). Both species have strong cold tolerance and overwinter underground as rhizomes. The lethal temperature at which 50% of rhizomes were killed in a freezing experiment ranged from −3.4°C to −6.3°C for both species [44]. Therefore, TTCS was set to −5°C and THCS to a very low accumulation rate below this threshold air temperature because of the expectation that rhizomes would be insulated by plant litter and snow pack in colder areas. DTCS was adjusted downward slightly from the temperate template (15°C) to 12°C for *M. sacchariflorus* and 14°C for *M. sinensis,* with a very low accumulation rate (DHCS; Table 1) because both species have high cold tolerance. DTCS was lower for *M. sacchariflorus* than for *M. sinensis* because native distribution records show *M. sacchariflorus* persisting at slightly higher latitudes.

There has been little investigation of heat stress effects on the growth of *Miscanthus*. Therefore, heat stress parameters (heat stress threshold, TTHS, and heat stress rate, THHS) were adjusted based on native distribution records for the species. TTHS was set to begin accumulating at or above the upper optimal growth temperature for *M. sacchariflorus* and *M. sinensis*, respectively, with a high accumulation rate (THHS). TTHS was higher and THHS was slightly lower for *M. sinensis* than for *M. sacchariflorus* to account for the former species’ more tropical native distribution.

*Soil moisture and dry/wet stress:* Limiting low soil moisture (SMO) and lower optimal soil moisture (SM1) were set according to the temperate template. Upper optimal soil moisture (SM2) and limiting high soil moisture (SM3) were set according to the wet-tropical template for *M. sinensis*. In comparison, SM2 and SM3 were reduced slightly for *M. sacchariflorus* in conjunction with the wet stress parameters to limit its potential distribution from occurring widely in wet tropical areas.

The dry stress threshold (SMDS) and dry stress rate (HDS) were set to accommodate the high drought tolerance of both species [45]. Both species were assigned an SMDS of 0.1, which is close to the minimum moisture content at which plants can extract water from the soil [46], [47]. HDS was set at a moderate rate, given that the hybrid can recover from short-term (30 days) but not long-term (60 days) drought [48].

Both *M. sacchariflorus* and *M. sinensis* grow well in saturated areas such as drainage ditches (HAH, personal observation), but not when submerged such as in streams [49], [50]. Therefore, the wet stress threshold (SMWS) and wet stress rate (HWS) were set so that the species would grow in wet areas but would experience a high rate of stress accumulation. Parameters for *M. sacchariflorus* were adjusted to restrict its southerly distribution.

*Hot-wet stress:* A hot-wet stress was added to the model for *M. sacchariflorus* to exclude its distribution from tropical equatorial areas of southeastern Asia-Pacific [37]. No hot-wet stress was used for *M. sinensis.*

### Sensitivity to Model Parameters

Once the models were validated, we performed a sensitivity analysis for each species to determine the response of the modelled distributions to small changes in parameter values. Each parameter was adjusted upward or downward while holding all other parameters constant, and the resulting EI values for the global distribution were generated in CLIMEX. Rate and soil moisture variables were adjusted by 10%; temperature variables were adjusted by 1°C (D. Kriticos, *personal communication*). EI was divided into five classes of suitability for species growth for further analysis: 0, unsuitable; 1–10, marginal; 10–20, suitable; 20–30, favourable; and >30, highly favourable [51], [34].

EI values from each iteration of the sensitivity analysis were compared with those of the original model to determine the change in area for each EI class. To do this, EI data generated by CLIMEX were reprojected to a cylindrical equal area projection in ArcGIS 10.1, masked to land area, and a nearest neighbour, inverse distance weighting interpolation was performed so that each grid cell represented an equal amount of area (50×50 km). The resulting grid cells were then reclassified corresponding to the five EI classes and the number of cells counted to determine the total area in each class. We then computed the proportional change in area from the original model for each EI class. Sensitivity was evaluated as a greater proportional change in area than in parameter value.

### Climate Change Scenarios

To compare the species’ potential distributions under future climate change predictions, and to assess the sensitivity of these potential distributions to model selection and emissions assumptions, we used two general circulation models with two emissions scenarios (A2 and B1). We used the Bergen Climate Model 2.0 (BCM) from the Bjerknes Center for Climate Research, and the Coupled Global Climate Model 3 (CGCM3-T63) from the Canadian Center for Climate Modeling and Analysis [52]. The selected emissions scenarios represent comparatively low (B1) and high (A2) future greenhouse gas concentrations, thus spanning the likely range of probable future conditions [53].

Climate projection data for each model–scenario combination were input into CLIMEX as the 30-yr mean for three standard time periods: baseline (1971–2000), 2050s (2041–2070), and 2080s (2071–2100). First, monthly means of maximum temperature, minimum temperature, and relative humidity, and daily means of precipitation for the world (excluding Greenland, Antarctica, and the Arctic) were downloaded from the Canadian Climate Change Scenarios Network (CCCSN: http://www.cccsn.ec.gc.ca/?page=dd-gcm) for each model–scenario combination. The 30-yr means were then calculated to obtain a single value for each climate variable for each 2.75°×2.75° grid cell. Because CLIMEX requires monthly climate data, the average daily precipitation data were multiplied by the number of days in the month to produce monthly means. CLIMEX also requires two relative humidity values, RH% at 0900 h and at 1500 h. Although only mean monthly values are available from the CCCSN, there is a strong diurnal cycle in RH%, with maximum values at night and minimum values in mid-afternoon. Daily mean values are therefore reasonably close to observed values at 0900 h and were considered RH% at 0900 h. RH% at 1500 h was estimated using the CLIMEX method of multiplying the 0900 h value by 0.85 [34], [31].

Using these model–scenario data, the CLIMEX bioclimatic model was run for each species. EI values were obtained for a total of 20 model runs ([circulation model baseline + two scenarios × two future time periods] × two models × two species). The EI data were reprojected into a cylindrical equal area projection, masked to land area, and interpolated using inverse distance weighting of the three nearest points in ArcGIS 9.3. The areas of suitable climate under baseline and future conditions were calculated by reclassifying the raw EI data into the five EI classes. Projected changes in the potential distributions of each species were determined by overlaying future and baseline projections.

For each species and time, areas of agreement between models or scenarios were determined by overlaying projected distributions based on the two models for a given scenario or the two scenarios for a given model, respectively. To quantify the similarity between pairs of projected potential distributions, we calculated a simple index of agreement by dividing the area where the two projections agree about the potential presence of *Miscanthus* (all area classified as suitable, favourable, and highly favourable, or EI >10) by the area where the two projections disagree. The higher the value, the more similar the two projections are. Values >1.0 indicate that the models agree more than they differ; values <1.0 indicate more disagreement than agreement.

## Results

**Figure 1:**
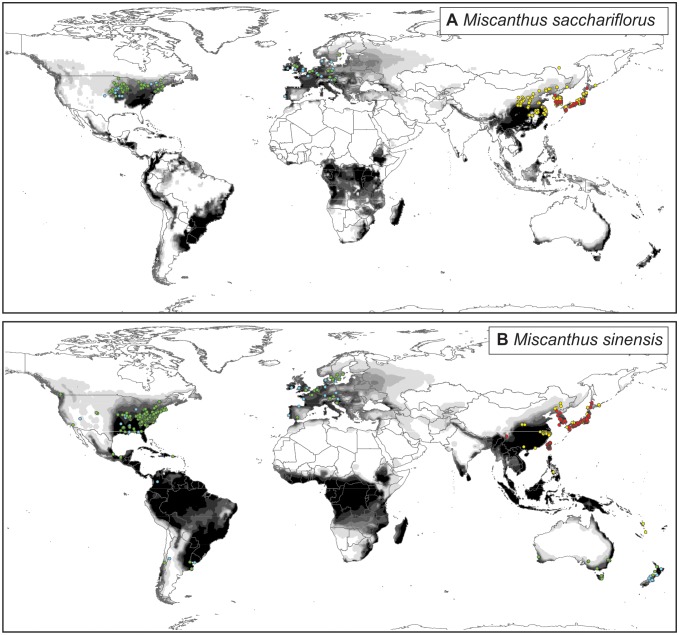
Observed (circles) and modelled (shaded) native and introduced plant distributions. Circles indicate native (red), native inferred (yellow), introduced (green), and introduced inferred (blue) geolocations. The density of shading represents the bioclimatic suitability of a region for plant population persistence, with white areas indicating no suitability, lighter grey areas indicating low suitability, and darker grey areas indicating high suitability. (a) *Miscanthus sacchariflorus*. Geolocations: native (n = 94), native inferred (n = 171), introduced (n = 119), and introduced inferred (n = 43). (b) *Miscanthus sinensis.* Geolocations: native (n = 335), native inferred (n = 52), introduced (n = 297), and introduced inferred (n = 81).

Occurrence records indicate that both the native and introduced distributions of *M. sacchariflorus* are less widespread than those of *M. sinensis* (Fig. 1). In the native range, both species occur throughout Japan, Korea, and south-central and eastern China, and in parts of northern and northeastern China. However, *M. sinensis* extends further south and east into Taiwan, the Philippines, and Vanuatu [37] (Fig. 1). In the introduced range, *M. sacchariflorus* occurs mainly in northwestern Europe, Denmark, Sweden, northeastern United States, and southeastern Canada. *M. sinensis* has been introduced to these areas as well as southeastern and parts of western United States, Mexico, Puerto Rico, Colombia, Chile, Argentina, Uruguay, southern Australia, Tasmania, and New Zealand.

For the *M. sacchariflorus* model, 100% of given native, 90.1% of inferred native, 89.1% of given introduced and 97.7% of inferred introduced occurrence records are in areas deemed suitable, favourable, or highly favourable (EI>10). Zero and 9.4% of given and inferred native, and 10.9% and zero of given and inferred introduced occurrences are in marginal areas. One inferred native record (0.6%) and one inferred introduced record (2.3%) are in areas deemed unsuitable. For the *M. sinensis* model, 100% of given native, 94.2% of inferred native, 99.7% of given introduced, and 97.5% of inferred introduced occurrences are in areas deemed suitable, favourable, or highly favourable. Zero and 5.8% of given and inferred native, and 2.0% and 2.5% of given and inferred introduced occurrences are in marginal areas. No observed occurrences are in areas deemed unsuitable. The models indicate that *M. sinensis* has a wider potential global distribution than does *M. sacchariflorus* (Fig. 1).

### Sensitivity to Model Parameters

The modelled distributions differed in their sensitivity to a ±10% or 1°C change in parameter values (Table 2). Eight of 22 (36%) parameters for *M. sacchariflorus* and 5 of 19 (26%) parameters for *M. sinensis* showed sensitivity in at least one EI class. Models for both species were very sensitive to changes in heat stress temperature threshold (TTHS) and upper optimal and limiting temperatures (DV2, DV3) and were mildly sensitive to changes in upper optimal and limiting moistures (SM2, SM3). In addition, the *M. sacchariflorus* model was highly sensitive to an increase in dry stress rate (HDS) and a decrease in hot-wet temperature threshold (TTHW), and mildly sensitive to a decrease in wet stress moisture threshold (SMWS).

**Table 2:** Sensitivity analysis results for *Miscanthus sacchariflorus* and *M. sinensis* indicating the proportional change in area from that of the baseline model global distribution for each category of ecoclimatic index (EI) modelled using CLIMEX software.

	*Miscanthus sacchariflorus*						*Miscanthus sinensis*					
Parameter	Value^a^	Proportional change in area from baseline (%)					Value^a^	Proportional change in area from baseline (%)				
		EI = 0	EI = 1–10	EI = 10–20	EI = 20–30	EI = 30–100		EI = 0	EI = 1–10	EI = 10–20	EI = 20–30	EI = 30–100
DV0	4 (20)	−0.61	0.98	1.17	−1.08	2.50	4 (20)	−0.71	0.36	1.81	−0.76	1.09
	6 (20)	0.69	−1.39	−1.28	2.13	−3.01	6 (20)	0.85	−1.16	−1.75	1.88	−1.30
DV1	14 (6.7)	−0.14	−2.13	0.19	−1.66	3.68	14 (6.7)	−0.19	−2.45	0.79	−0.88	1.55
	16 (6.7)	0.12	2.15	0.11	2.67	−4.32	16 (6.7)	0.19	2.12	−0.79	2.61	−1.83
DV2	27 (3.6)	0.06	**5.31**	**9.94**	−0.27	−**11.68**	29 (3.3)	0.11	1.77	**8.97**	**16.08**	−**7.08**
	29 (3.6)	−0.07	−**5.62**	−**7.63**	−2.98	**12.25**	31 (3.3)	−0.13	−1.01	−**3.92**	−**13.90**	**5.10**
DV3	31 (3.1)	0.54	**10.64**	−0.84	−**7.20**	−**9.72**	34 (2.9)	0.26	0.46	**5.41**	**5.22**	−**3.28**
	33 (3.1)	−0.15	−**5.80**	−**4.26**	3.08	**7.80**	36 (2.9)	−0.12	−0.46	−1.62	−**5.13**	2.11
SM0	0.225	−0.77	1.62	1.62	−0.10	2.03	0.225	−1.46	2.97	3.59	−1.03	1.11
	0.275	0.71	−1.43	−1.67	0.57	−2.10	0.275	1.41	−2.80	−3.76	1.52	−1.17
SM1	0.72	−0.28	−3.06	−0.42	−2.27	6.18	0.72	−0.54	−3.30	1.42	−4.28	3.36
	0.88	0.24	4.14	−1.59	2.13	−5.68	0.88	0.38	4.48	−3.69	5.13	−3.26
SM2	1.08	0.03	4.32	7.60	7.37	−**12.94**	0.9	0.00	1.13	2.74	**10.08**	−3.65
	1.32	−0.01	−3.89	−6.71	−4.50	**10.39**	1.1	−0.01	−0.91	−3.23	−8.07	3.18
SM3	1.62	0.17	7.86	7.60	1.22	−**14.21**	2.25	0.00	1.01	4.39	**13.26**	−4.76
	1.98	−0.05	−5.12	−5.40	1.93	7.81	2.75	−0.01	−0.50	−2.77	−7.37	2.71
TTCS	−6 (20)	0.76	−1.86	−2.26	−0.91	−0.83	−4 (20)	−1.10	2.60	2.67	0.88	0.26
	−4 (20)	−0.78	2.32	2.42	0.37	0.67	−6 (20)	1.00	−1.66	−4.25	−0.36	−0.25
THCS	−0.00022	0.66	−2.56	−1.45	−0.44	−0.31	−0.00022	0.81	−1.96	−2.80	−0.24	−0.08
	−0.00018	−0.83	3.34	2.03	0.44	0.19	−0.00018	−1.07	3.18	2.61	0.24	0.11
DTCS	11 (8.3)	−0.36	0.95	1.20	0.10	0.40	13 (7.1)	−0.42	0.61	1.48	−0.21	0.31
	13 (8.3)	0.38	−1.15	−0.53	−0.41	−0.57	15 (7.1)	0.44	−0.63	−1.78	0.03	−0.23
DHCS	−0.000055	0.26	−0.69	−0.39	−0.17	−0.55	−0.000055	0.33	−0.23	−1.62	−0.12	−0.17
	−0.000045	−0.27	0.71	0.95	0.07	0.31	−0.000045	−0.42	0.68	1.32	−0.12	0.28
TTHS	31 (3.1)	**8.79**	−**13.96**	−**22.28**	−**21.78**	−**13.97**	35 (2.8)	2.29	−**3.12**	−2.01	−**7.37**	−1.03
	33 (3.1)	−**8.15**	**26.66**	**16.04**	**8.12**	**8.21**	37 (2.8)	−1.82	**3.30**	**3.99**	**5.01**	0.11
THHS	0.054	−0.21	0.46	0.72	0.30	0.21	0.045	−0.05	0.10	0.16	0.09	0.00
	0.066	0.18	−0.46	−0.47	−0.41	−0.14	0.055	0.08	−0.10	−0.43	−0.06	0.00
SMDS	0.09	−0.12	−0.15	−1.64	0.98	1.39	0.09	−0.42	0.28	−2.84	0.55	1.25
	0.11	−0.36	−1.91	−6.35	−0.91	8.48	0.11	0.43	−0.02	1.39	0.21	−1.25
HDS	−0.022	−0.36	−1.91	−6.38	−0.88	8.48	−0.022	0.15	0.45	0.53	0.09	−0.66
	−0.018	−**17.00**	**88.48**	6.10	7.51	5.97	−0.018	−0.25	0.05	−1.65	0.76	0.68
SMWS	1.62	1.03	−2.96	−**10.05**	−2.71	4.37	2.7	0.01	0.23	−0.20	0.00	−0.08
	1.98	−0.63	−2.77	−5.35	0.57	9.55	3.3	−0.01	0.02	−0.03	0.03	0.02
HWS	0.018	−0.39	−2.08	−6.04	−0.78	8.57	0.045	0.00	0.00	−0.03	0.00	0.01
	0.022	−0.32	−1.94	−6.52	−1.05	8.45	0.055	0.00	0.00	0.00	0.00	0.00
TTHW	30 (3.2)	**3.67**	−**5.43**	−**6.91**	−**9.77**	−**7.37**						
	32 (3.2)	−1.15	2.87	**3.93**	0.95	1.12						
MTHW	0.9	0.14	−0.05	−0.58	−0.07	−0.40						
	1.1	−0.19	0.52	0.11	0.51	0.28						
PHW	0.009	−0.09	0.21	0.28	0.10	0.14						
	0.011	0.05	−0.02	−0.22	0.00	−0.15						
PDD	540	−0.60	−0.53	−6.35	−0.95	8.52	540	−0.27	1.21	0.03	0.00	0.00
	660	−0.13	−3.11	−6.74	−0.95	8.52	660	0.27	−1.14	−0.16	0.00	0.00

a Parameter value used in the sensitivity analysis. The percent change from the original model parameter value was 10%, except for temperature variables, which were changed by 1°C and for which percent change is indicated in parentheses. Changing temperature variables by 10% gave qualitatively similar results (data not shown).

Parameter values were each decreased and increased by 10% from the baseline value (Table 1) unless indicated otherwise^a^. Changes in area that exceed the percent change in parameter value are shown in boldface font; positive and negative proportions respectively indicate increases and decreases in area.

Sensitive temperature parameters all related to upper temperatures and heat tolerance thresholds (Table 2). A decrease in the upper temperature parameters tended to decrease EI towards less favourable values, whereas an increase tended to increase EI towards more favourable values for both species. The heat stress temperature threshold followed a similar pattern. The *M. sacchariflorus* model was more sensitive than the *M. sinensis* model to changes in these parameters. For *M. sacchariflorus*, a decrease in the hot-wet temperature threshold also decreased EI towards less favourable values, whereas an increase had only a small effect.

Sensitive soil moisture parameters all related to upper soil moistures and the wet stress threshold. Changes in upper soil moisture parameters had weaker effects than for temperature. A decrease in the upper soil moisture parameters tended to decrease the most favourable EI values but had little effect on the unsuitable climate area. An increase in these parameters had little effect, except for slight increases in EI towards more favourable values for *M. sacchariflorus*. For *M. sacchariflorus*, a decrease in the wet stress moisture threshold tended to decrease the suitable climate area.

The only rate parameter that exhibited sensitivity was the dry stress rate for the *M. sacchariflorus* model. An increase in this parameter increased EI values from unsuitable towards more favourable values, with the largest change in marginal climate area (Table 2).

### Range Shifts Under Climate Change Projections

**Figure 2:**
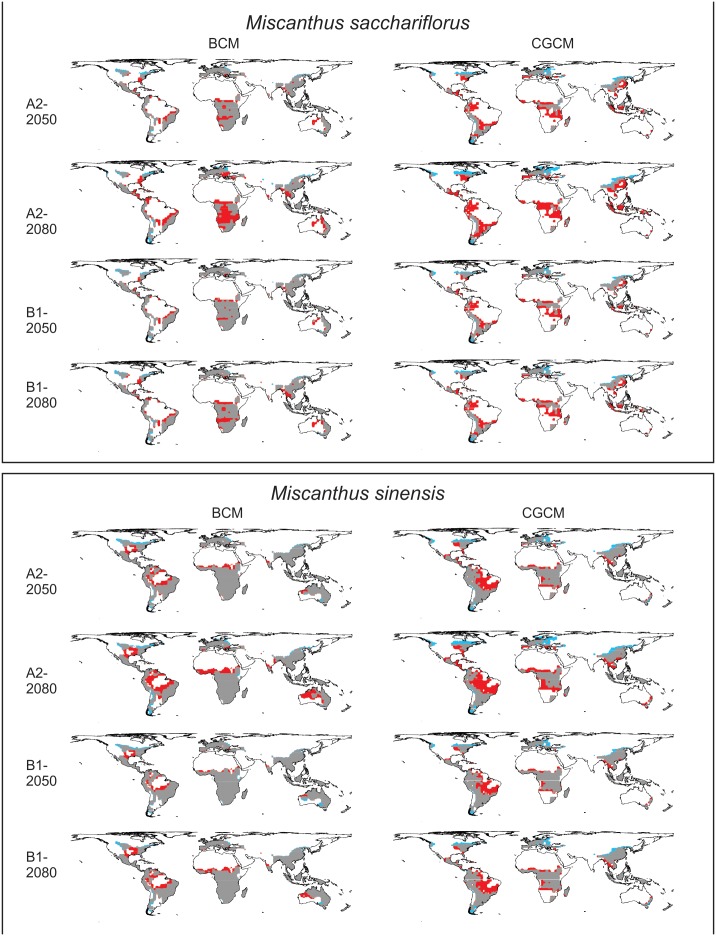
The potential future distribution of *Miscanthus sacchariflorus* and *M. sinensis* (for all area classified as suitable, favourable, and highly favourable, or EI >10) as modeled by CLIMEX under the BCM and CGCM models run using the A2 and B1 emissions scenarios. All potential ranges are shown relative to their baseline potential distributions. Areas shown in red indicate area where the range is projected to contract; grey indicates the species are potentially present under both baseline and future conditions; and blue indicates areas of range expansion.

Projected changes in the potential area occupied by *M. sacchariflorus* and *M. sinensis* are generally large, ranging from global decreases in potential area by 2080 of 4 to 6%, depending on the species, model, and scenario chosen (Table 3, World; Fig. 2). In all cases, the area of climatically suitable locations (EI >10) continues to decrease over time.

**Table 3:** Percent change in climatically suitable area for the World and for North America projected using the BCM and CGCM models and the A2 and B1 emissions scenarios relative to the baseline projections.

		World						North America					
Model	Year, scenario	EI 0–1	EI >1–10	EI >10–20	EI >20–30	EI >30	EI >10	EI 0–1	EI >1–10	EI >10–20	EI >20–30	EI >30	EI >10
		*Miscanthus sacchariflorus*						*Miscanthus sacchariflorus*					
**BCM**	2050 (A2)	+4.1	+6.9	+15.3	−1.7	−20.6	−**10.4**	−1.6	+1.5	+15.4	+74.5	−32.4	**+3.9**
	2080 (A2)	+12.4	+14.7	+8.1	−31.8	−38.6	−**28.9**	−4.4	+18.5	−2.7	+16.7	−18.8	−**6.7**
	2050 (B1)	+3.2	+2.1	+12.6	−0.2	−14.6	−**6.9**	−1.1	−0.3	+6.6	+58.6	−18.4	**+4.4**
	2080 (B1)	+6.5	+5.4	+9.3	−4.6	−24.3	−**14.3**	−1.1	+4.3	+4.8	+49.8	−26.2	−**1.4**
**CGCM**	2050 (A2)	+8.7	+7.4	−6.3	−12.0	−58.2	−**31.1**	−7.2	+32.8	+27.0	+5.0	−51.5	**+0.6**
	2080 (A2)	+13.0	+10.0	−11.8	−41.7	−71.9	−**46.1**	−13.9	+55.9	+47.7	+21.0	−70.1	**+9.9**
	2050 (B1)	+7.0	+6.4	−9.7	−9.9	−44.3	−**25.4**	−6.5	+26.0	+23.0	+18.2	−42.3	**+5.0**
	2080 (B1)	+8.6	+6.5	−3.8	−13.9	−57.5	−**30.5**	−6.7	+27.2	+27.6	+23.8	−59.9	**+4.3**
		***Miscanthus sinensis***						***Miscanthus sinensis***					
**BCM**	2050 (A2)	+4.0	+8.3	−7.4	−14.4	−5.0	−**6.9**	+1.9	−2.8	−6.5	+23.1	−7.1	−**1.9**
	2080 (A2)	+9.7	+19.1	−8.5	−32.4	−14.9	−**16.5**	−5.4	+28.9	−22.0	+10.3	−9.3	−**11.4**
	2050 (B1)	+1.8	+4.9	+0.7	−11.4	−2.7	−**3.5**	+1.6	+1.8	−11.4	+15.7	−6.8	−**5.0**
	2080 (B1)	+5.1	+12.8	−12.9	−16.1	−6.9	−**9.4**	+0.0	+17.9	−34.6	+17.4	−6.3	−**14.3**
**CGCM**	2050 (A2)	+8.9	−5.6	+8.3	−0.4	−24.5	−**13.4**	−8.2	+24.3	+13.0	+61.5	−42.1	**+5.3**
	2080 (A2)	+10.9	+6.9	+15.6	+5.9	−42.2	−**21.6**	−17.2	+44.2	+44.7	+101.6	−71.5	**+18.2**
	2050 (B1)	+7.1	−4.1	+6.5	−3.3	−19.0	−**10.9**	−6.7	+14.5	+5.1	+68.0	−22.3	**+9.9**
	2080 (B1)	+8.0	−5.2	+10.3	−1.0	−22.7	−**12.0**	−9.4	+22.0	+17.6	+78.0	−38.6	**+12.2**

The column EI>10 (bold) indicates a composite analysis combining all area classified as suitable, favourable, and highly favourable for each species.

Limiting this analysis to North America reveals some important regional differences (Table 3, North America). Projected percent changes in climatically suitable area are smaller for North America than for the world and are often different in sign. The direction of change also differs between the two models: For both species by 2080, regardless of scenario, the BCM projects reductions in the total suitable area and the CGCM projects increases in suitable area. There are also large changes in the relative proportions of habitat categories. In all projections, including those in which the area of climatically suitable habitat increases, highly favourable area (EI >30) decreases and favourable area (EI 20–30) increases.

Comparing the future potential distributions of *Miscanthus* species to their baseline potential distributions provides an indication of how rapidly shifts in suitable range might occur. This analysis indicates that projected suitability shifts are moderately large for both species and under both emissions scenarios (Table 4). The smallest projected shifts in suitable range occur under the B1 emissions scenario, with *M. sacchariflorus* projected to have larger shifts than *M. sinensis*. The mean overlap between the baseline and 2080s potentially suitable areas is only 61% for *M. sacchariflorus* and 78% for *M. sinensis*. Very little range contraction is projected to occur in the native range, except for some projections for *M. sacchariflorus.* In contrast, range expansion is projected in northern parts of the native range for both species. In the non-native range, contraction is projected mainly in areas where the species have not yet been introduced such as South America, Africa, and parts of Australia for both species, as well as islands of Southeast Asia for *M. sacchariflorus*. Contraction of the potential range is also projected to occur in southern parts of the United States, but more so for *M. sinensis* than for *M. sacchariflorus*. The majority of range expansion is projected to occur in northern areas of North America, eastern Europe, and Scandinavia.

**Table 4:** Projected changes in the global climatically suitable area for *Miscanthus sacchariflorus* and *M. sinensis* under the BCM and CGCM models run using the A2 and B1 scenarios.

Model	Year, scenario	Expansion^a^ (km×10^6^)	Contraction (km×10^6^)	No change (km×10^6^)	Overlap^b^ (%)
		*Miscanthus sacchariflorus*			
**BCM**	2050 (A2)	1.54	5.74	34.81	86%
	2080 (A2)	2.28	13.99	26.56	65%
	2050 (B1)	1.10	3.88	36.67	90%
	2080 (B1)	1.35	7.16	33.38	82%
**CGCM**	2050 (A2)	3.08	11.94	16.51	58%
	2080 (A2)	5.08	18.20	10.25	36%
	2050 (B1)	2.33	9.56	18.89	66%
	2080 (B1)	2.73	11.41	17.04	60%
		***Miscanthus sinensis***			
**BCM**	2050 (A2)	2.08	5.85	48.85	89%
	2080 (A2)	2.78	11.79	42.91	78%
	2050 (B1)	1.95	3.85	50.84	93%
	2080 (B1)	1.60	6.72	47.98	88%
**CGCM**	2050 (A2)	3.68	9.36	33.10	78%
	2080 (A2)	6.01	15.17	27.29	64%
	2050 (B1)	2.84	7.47	35.00	82%
	2080 (B1)	3.51	8.60	33.86	80%

a Potential future suitable area is defined as EI>10.

b Overlap is calculated as the portion of the baseline range that overlaps with the projected range.

### Sensitivity of Results to Choice of Climate Model and Scenario

**Figure 3:**
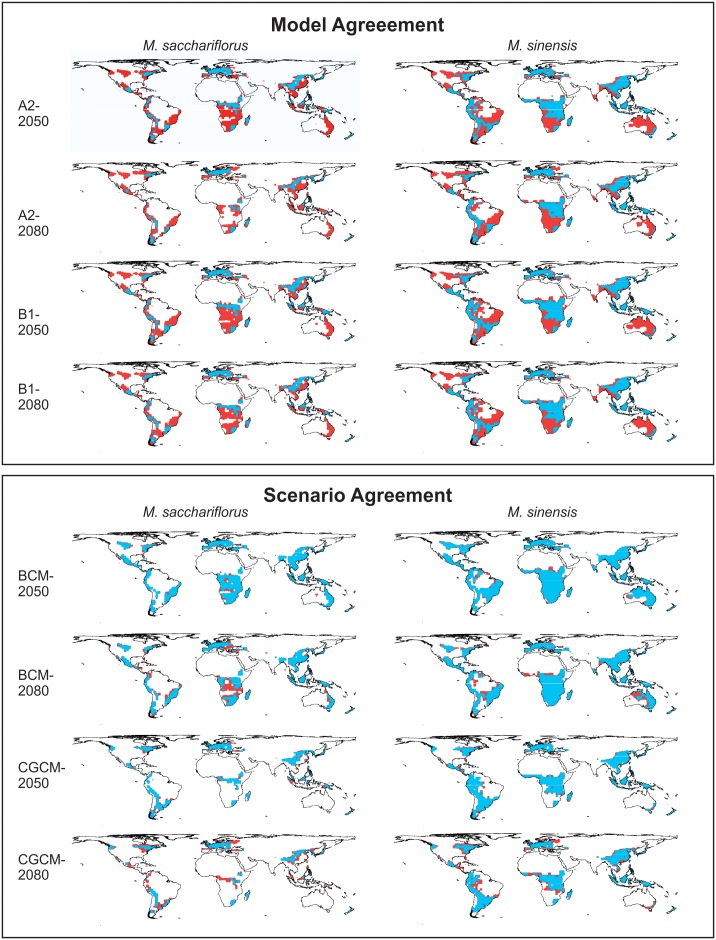
Areas of agreement and disagreement in the projected potential distribution of *Miscanthus sacchariflorus* and *M. sinensis* (all area classified as suitable, favourable, and highly favourable, or EI >10) as modeled by CLIMEX. The upper model shows areas of agreement between the BCM and CGCM models for a given scenario and year. The lower panel shows areas of agreement between the A2 and B1 scenarios for a given model and year. Areas shown in blue indicate suitable climate under both projections; areas shown in red indicate that only one of the two projections predicts suitable climate.

By overlaying the results for the different models and scenarios, we can identify patterns of agreement and disagreement between the various projections (Fig. 3). This analysis helps to identify changes in the suitable range that are highly likely (e.g., robust to the selection of model or scenario), versus those that are more speculative (e.g., those that differ depending on the model or scenario chosen). This analysis also allows us to identify whether the projections differ because of differences between the climate models or between the scenarios. These results indicate that the choice of climate model accounts for more difference in the results than the choice of scenario and that the two *Miscanthus* species differ in their sensitivity to the selection of model and scenario (Table 5). For the model sensitivity analysis, agreement values are all either <1.0 (greater area of disagreement than agreement between models; five out of eight comparisons), or very slightly >1.0 (maximum value 1.22). For the scenario sensitivity analysis, all agreement values are substantially >1.0. Different scenarios are more similar within models than are the same scenarios between models for these species.

**Table 5:** The index of agreement^a^ between different models run under the same scenario and between different scenarios run within the same model, for projected distributions of *M. sacchariflorus* and *M. sinensis* in 2050 and 2080.

*Model agreement:*	*2050*	*2080*
*M. sacchariflorus*–A2	0.58	0.44
*M. sacchariflorus*–B1	0.64	0.63
*M. sinensis*–A2	1.17	0.96
*M. sinensis*–B1	1.21	1.22
***Scenario agreement:***		
*M. sacchariflorus*–BCM	13.68	3.23
*M. sacchariflorus*–CGCM	5.28	1.33
*M. sinensis*–BCM	16.35	6.25
*M. sinensis*–CGCM	11.74	3.25
***Model average:***	0.90	0.81
***Scenario average:***	11.76	3.51

a The index is calculated as the ratio of the area of overlap between the two projections to the area of non-overlap.

## Discussion

### Potential Global Distribution

Niche and bioclimatic envelope models in general provide a coarse-scale indication of areas where the climate might be suitable for the species of interest to establish. The limitations of such models are well discussed elsewhere [34], [54], [55], [56]. Here, we note two associated factors that require consideration in interpreting our models: the species’ ecology and their likely non-equilibrium distribution in the introduced ranges.

Little is known of the species’ comparative ecology in the native and introduced ranges because most research to date has focused on determining optimal conditions for agricultural production (but see [57], [42], [50]). Parameterizing a model based solely on the native range distribution assumes similar biotic interactions in the introduced and native ranges. However, potential release from suppressive interactions such as competition, herbivory, parasitism, and disease (e.g., [58], [59], [60]), or a lack of mutualist organisms (e.g., [61]) could result in different realized distributions or niche shifts in the introduced compared to the native range (e.g., [62], [63], but see [64], [65]). We parameterized our model using both the native range distribution and some physiological data, which could improve model estimations compared to strictly correlative methods [66], [67]. Model validation using occurrences in the introduced range indicates that the models fit the current introduced distributions well. Determining whether an introduced species will establish beyond the modelled range requires further fine-scale assessment and/or field experiments [54], [68].

It is highly likely that *M. sinensis* and *M. sacchariflorus* are still spreading in the introduced ranges. Indeed, the species only became naturalized in North America in the mid-1900s [12], [11], and time since introduction is a well-known correlate of plant escape and abundance in the introduced range (e.g., [69], [70]). The bioclimatic models suggest that there are additional moderate to large amounts of climatically suitable area in Central America, South America, and Africa, as well as small parts of Australia and New Zealand, where these species have not yet established. The likelihood of spread beyond the modelled potential distributions is unknown, but given the increase in cultivation of these species as both horticultural and agricultural materials, which reduces dispersal limitations, and the potential for plant breeding programs to introduce new genetic material with a wider range of trait variation, proliferation within and beyond the introduced area should be monitored closely.

According to the native distributions and our models, *M. sinensis* has a wider range and greater climatically suitable area than *M. sacchariflorus* [37] (Fig. 1). Although these characteristics could make *M. sinensis* attractive for cultivation across a wide area, species that have wider native distributions and occur in more habitats and climate zones are likely to be more successful as invaders, regardless of other biological traits [71]. Thus, *M. sinensis* might also have greater potential to become a weedy or invasive plant than does *M. sacchariflorus*. Rather than developing one or two cultivars suitable for widespread production, a best management practice might be to develop regionally restricted cultivars to minimize widespread escape and invasion of novel species from the agriculture and horticulture trades.

### Consequences of Parameter Sensitivity

The smaller native distribution and therefore narrower environmental tolerances of *M. sacchariflorus* likely contribute to its greater sensitivity to changes in model parameters than for *M. sinensis*. If the extent of the native distribution is correlated with model sensitivity across a range of species, this would have implications for modelling and interpreting models for both invasive species and rare species of conservation concern. For potential invaders, this might mean that some priority is given to species with wide distributions. For rare species, i.e., those with small native distributions that are not due to anthropogenically caused local extinctions, obtaining accurate model results could require greater accuracy in parameter estimation.

The two *Miscanthus* species models showed sensitivity to similar parameters, which might not be surprising, given that their distributions overlap in temperate areas. However, the main sets of parameters exhibiting sensitivity were those for which there are the least data. The most sensitive parameters were related to upper temperatures and heat tolerance, but most studies of temperature-related growth for these species have examined cold tolerance because of interest in their cultivation at northern latitudes (e.g., [39], [38]). Physiological heat thresholds remain to be explored for these species to improve the confidence of lower-latitude thresholds for growth in the northern hemisphere, where they have been introduced, as well as potential range contractions at lower latitudes under climate change.

Similarly, although weakly sensitive and thus potentially of lesser importance the upper temperature parameters, upper soil moistures and moisture tolerance have rarely been examined. Most studies of soil moisture effects for these species examine drought, rather than saturation (e.g., [45], but see [50]). The accuracy of these parameter estimates could be important in predicting potential invasion of these species into drainage ditches, riparian areas, and wetlands.

Most stress rate parameters were relatively insensitive to changes in value. CLIMEX determines stress as the annual exponential accumulation of weekly population reduction when a stress threshold is exceeded [34]. Stress accumulation rate is difficult to estimate empirically without extensive field or laboratory trials under various stress thresholds, and the magnitude of the accumulation rate could depend on the threshold value chosen; for many species, few data of this type likely exist [72]. However, the minimal sensitivity of the stress rate parameters implies that their accuracy is less influential than that of other, more easily estimated parameters.

Two previous tests of sensitivity using CLIMEX have some similarities. A study of the invasive tropical/subtropical shrub *Lantana camara* identified model sensitivity to limiting low and high temperatures and limiting low soil moisture [27]. A study of the invading pathogen *Phytophthora ramorum* identified model sensitivity to optimal high temperature and limiting and optimal low soil moisture [26]. However, neither study tested model sensitivity to the stress rate parameters. Nevertheless, both our and their models show high sensitivity to some of the limiting upper or lower temperature and moisture parameters. Sensitivity analyses should be performed for additional species to determine whether some parameters are consistently more sensitive than others. If sensitivity to specific parameters is consistent within biomes and species types (forb, shrub, etc.), researchers could focus their efforts on measuring those specific environmental tolerances to maximize model estimation accuracy.

### Future Climate Projections

Although the climatically suitable area for the two *Miscanthus* species is projected to decrease globally with climate change, areas of North America, eastern Europe, and Scandinavia are projected to experience some future increase in suitable climate. This could be beneficial for cultivating these species as bioenergy crops in these regions if suitable habitat is available. However, it could also place these regions at greater risk of invasion through increases in the area of suitable climate outside of cultivation. These regions, in particular, are projected to be future hotspots of invasion for 99 of the worst invaders globally [73]. Areas of range contraction for the two *Miscanthus* species also coincide with areas where future invasion is projected to decrease [73].

Additionally, climate niche projections do not account for the potential that rapid evolution in introduced species and their recipient communities could allow species to become invasive beyond their current tolerances (e.g., [74], [75], [76], [77]). In these *Miscanthus* species, rapid evolution could be aided by the introduction of new horticultural genotypes from widely separated populations in the native range [78]. These species are obligate out-crossers [79], so isolated populations composed of a single clone do not produce seed. Introduction of different genotypes could increase the probability of sexual reproduction and long-distance spread via the wind-dispersed seed [80]. Similarly, if these plants are developed for biomass production, intensive plant breeding programs will aim to improve their performance under a variety of conditions, including resistance to pests and disease, drought and heat tolerance, cold tolerance, and possibly salinity tolerance [81]. These efforts will potentially expand the plants’ realized distributions as well as their invasive potential.

Variation in the area of future projected climate was greater among climate models than among emissions scenarios. This has also been found previously, when quantified, for both plant (e.g., [82], [83], [84]) and insect (e.g., [31], [32]) species under a number of different model-scenario combinations. Coupled with the observation that some currently observed climate changes might be greater than those predicted by even the highest emissions scenarios [85], this result suggests that future bioclimatic envelope model projections should focus efforts on increasing the number of models compared using one high-emissions scenario to develop composite projections of future suitable climate areas.
